# Supplementary material for: Identification and diagnostic evaluation of an aptamer targeting prostate-cancer-derived small extracellular vesicles
Source: Mol Ther Nucleic Acids. 2026 Jan 15;37(1):102836. doi: 10.1016/j.omtn.2026.102836 (PMC12870759; doi:10.1016/j.omtn.2026.102836)
Supplement: Document S1. Figure S1 and Tables S1–S5 [file mmc1.pdf]

OMTN, Volume *37*

Supplemental information

Identification and diagnostic evaluation of an aptamer targeting prostate-cancer-derived small extracellular vesicles

Ting Ding, Yue Li, Li Xue, Chaoliang Xiong, Lijuan Yu, Qian He, Jiayun Liu, Xiaoke Hao, and Dan Zhao

Supplemental materials and methods

sEVs isolation and characterization

The freshly collected samples were sequentially centrifuged at 300 g for 10 minutes to remove cells, at 2000 g for 20 minutes to eliminate cell debris, at 10000 g for 30 minutes to isolate large extracellular vesicles (lEVs), and finally at 120,000 g (Optima XPN-90, Type 45 Ti rotor, Beckman Coulter, USA) for 2.5 hours to isolate small extracellular vesicles (sEVs). Post-ultracentrifugation, the pellet was resuspended in PBS and stored at -80°C until use. sEVs morphology was analysed using transmission electron microscopy (TEM) (Tecnai, USA). In brief, vesicles were loaded onto the glow-discharged 300-mesh copper grids (Electron Microscopy Sciences, Hatfield, PA) for 30 seconds, then washed by water twice, and further stained with 2% uranyl formate for 1 min. Negative stained sEVs were analyzed by digitization on a FEI Tecnai G2 F20 X-TWIN Transmission Electron Microscope at 120 kV. To examine sEVs size distribution and particle concentration, nanoparticle tracking analysis (NTA) was performed using a ZetaView instrument (Particle Metrix, Germany) with a dilution of 1:10000, and parameters as below: sensitivity:80%, Max Area: 1000, Min Bright 30, Min Area 10.

EV-SELEX

SEVs isolated from PCa cell lines were employed for positive selection, while those derived from normal immortalized prostate cell lines served as negative selection. An ssDNA 76 nt randomized oligonucleotide library and required primers were chemically synthesized by Sangon Biotech (Shanghai, China) and the library information was provided in Table S4. To maintain EV membrane integrity and aptamer active conformation (and provide a suitable physicochemical environment for their specific binding), the binding reaction between EVs and aptamers was performed in the following system: reaction buffer (1× PBS, 3 mM $MgCl_2$, 0.5% Pluronic® F127). A total of nine rounds of screening were conducted (detailed conditions in Table S1), the key steps were as follows: The DNA library was subjected to denaturation (95°C for 10 min) and renaturation (ice bath for 5 min). For each round, the renatured library was first incubated with negative selection EVs for 1 hour. Aptamers bound to the negative

selection EVs were removed by centrifugation following the addition of PEG8000. The supernatant was collected and then incubated with positive selection EVs for 1 hour. Subsequently, the EV-aptamer complexes were precipitated with PEG8000, and the aptamers associated with positive selection EVs in the pellet were eluted with ultrapure water by boiling water bath. The eluates were quantified by Q-PCR, and the positive eluate was amplified by emulsion PCR (ePCR). Single-stranded DNA was isolated by denaturing PAGE electrophoresis, followed by dialysis, concentration, and quantification using a microvolume UV spectrophotometer. The enriched library was then used as the template for the next round of selection.

Nanoflow cytometry

Nanoflow cytometry analysis was performed using a Flow NanoAnalyzer (NanoFCM, China). The gating strategy to identify aptamer-positive EVs was as follows. Filtered phosphate-buffered saline (PBS) was used to establish the background scatter signal and exclude non-EV particles. Unstained EVs (not incubated with fluorescent aptamers) served as the negative control to define the autofluorescence threshold for the FAM channel. Events exceeding these thresholds were considered specifically stained EV populations. For signal detection, the light emitted by individual nanoparticles or EVs was collected perpendicularly to both the laser beam and the sample stream by an infinity-corrected microscope objective. Two single-photon counting avalanche photodiodes (APDs) were used for the simultaneous detection of side scatter and green FL (Fam signal) of individual particles, respectively. The 488 nm laser power was fixed at 20mW and the SS decay at 10%. The threshold levels for both the peak height (a digital discriminator level set to 3 times the standard deviation of the background) and the peak width of 0.2 and 0.3 ms were set as the criteria for burst (or peak) identification of SSC and FL signal, respectively. For each burst that satisfied the criteria, the integrated number of photons (background subtracted) was stored as the burst area for the histogram or dot-plot construction. The sampling pressure was fixed at 1.0 kPa prior to acquisition and events were recorded for a duration of 1 min for each sample.

Supplemental Figures

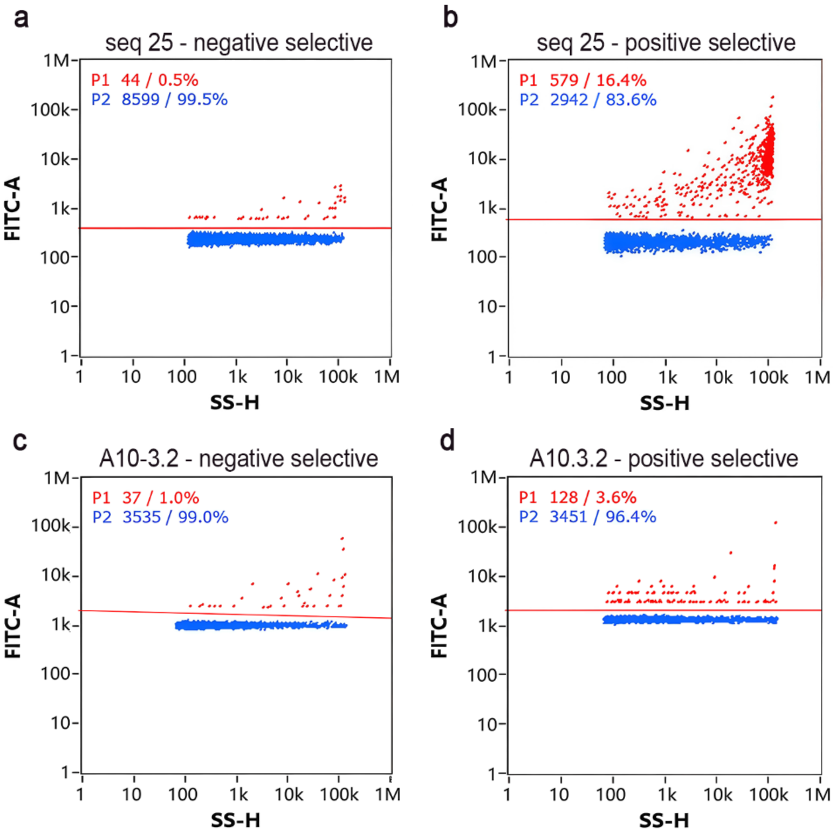

Figure S1. Comparison of binding specificity to PCa-EVs between seq25 and PSMA aptamer A10-3.2. a. Positive vesicles proportion of positive selection after co-incubation with seq 25 by Nanoflow. **b.** Positive vesicles proportion of negative selection after co-incubation with seq 25 by Nanoflow. **c.** Positive vesicles proportion of positive selection after co-incubation with A10-3.2 by Nanoflow. **d.** Positive vesicles proportion of negative selection after co-incubation with A10-3.2 by Nanoflow.

Table S1. Condition of EV-SELEX.

Round	Library	Negative selection	Positive selection
1	10μM, 137μL	0.6μg/μL, 50μl, 60min	0.5μg/μL, 60μl, 60min
2	700nM,150μL	0.6μg/μL, 50μl, 50min	0.5μg/μL, 60μl, 50min
3	560nM,100μL	0.6μg/μL, 50μl, 40min	0.5μg/μL, 60μl, 40min
4	400nM,150μL	0.6μg/μL, 50μl, 40min	0.5μg/μL, 60μl, 40min
5	300nM, 200μL	0.6μg/μL, 50μl, 30min	0.5μg/μL, 60μl, 30min
6	600nM,100μL	0.6μg/μL, 50μl, 30min	0.5μg/μL, 60μl, 30min
7	300nM,100μL+5ul, 1mg/ml salmon sperm DNA	0.6μg/μL, 50μl, 30min	0.5μg/μL, 60μl, 30min
8	300nM,100μL+10ul, 1mg/ml salmon sperm DNA	0.6μg/μL, 50μl, 30min	0.5μg/μL, 30μl, 30min
9	300nM,100μL+20ul, 1mg/ml salmon sperm DNA	0.6μg/μL, 50μl, 30min	0.5μg/μL, 30μl, 15min

Table S2. Sequencing results of the top 30 aptmers.

Aptmer name	Reads	Aerosol pollution	Enrichment ratio	Sequence
seq1-01	18726	68	2.380%	CACGCATAACTATCCACACCCGACCTCCGATCATGGCAGTTGCACTGTGTTATGCG
seq1-02	18230	54	2.319%	CACGCATAACTGTTCATGACACGTCACACTGCACCATCGATCGCATCGTGTTATGCG
seq1-03	16714	56	2.125%	CACGCATAACTCGACAACTGTCCGTCGACCGCTGGGGCATTCATGAGTGTTATGCG
seq1-04	16268	112	2.061%	CACGCATAACGCTCCATAGCATGGATCGCGCTGCCGGCACTTGTGTGTGTTATGCG
seq1-05	13545	39	1.723%	CACGCATAACCAAGCCGTAGTGTAGCGTAGTGTGTGTTTGCCCATGGTGTTATGCG
seq1-06	10370	158	1.303%	CACGCATAACGGGCCGGCTGTCGGATCCGTGCCGCGGAATTCATGTGTGTTATGCG
seq1-07	9576	80	1.211%	CACGCATAACGTCTCGCTCGCAGCGGACCCCTATCGGTGGCTCATTGGTGTTATGCG
seq1-08	9224	76	1.167%	CACGCATAACGAAGCATGCTAGGGATTCGCGCCGCGGCATTCATGTGTGTTATGCG
seq1-09	7997	47	1.014%	CACGCATAACCACCGACATCGCATGTCCACTTCAGTCGTCCTCGAGTGTTATGCG
seq1-10	7646	66	0.967%	CACGCATAACCGGTACAGCCAGGTAACGGCGCCGCGGCATTCATGTGTGTTATGCG
seq1-11	7409	44	0.940%	CACGCATAACTCCGCACAATTGCCTATGTGTTTGCCACCGGATGCAGTGTTATGCG
seq1-12	6527	41	0.827%	CACGCATAACGGACACCGTTCAAGGCCCTAGACAGCTACCTTTGAAGTGTTATGCG
seq1-13	6499	62	0.821%	CACGCATAACCGTGCATGGCATGTGCGCGGCCCGAGGCATTGATGTGTGTTATGCG
seq1-14	6252	76	0.788%	CACGCATAACCATGGGCCCGTCCCCGGCGAACAGATCGCATCTGGTGTGTTATGCG
seq1-15	6157	31	0.781%	CACGCATAACGACACCGTTGAACTGCTTCCACGAGCTTCCATTCAAGTGTTATGCG
seq1-16	6091	40	0.772%	CACGCATAACTGAGCACCTGTGAGCTCAACGCTGTGGCACTTGTCAGTGTTATGCG
seq1-17	6079	97	0.763%	CACGCATAACTGTCGGTTGCACAGACACTGGCATGGCCACTTGTAGGTGTTATGCG
seq1-18	5992	86	0.753%	CACGCATAACCCGGTCTCGCAGATCGGACGTTGTGACAGTTCTTGTGTGTTATGCG
seq1-19	5837	46	0.739%	CACGCATAACTGGCTAGATCGCATCGCAGTTCATCCCTGGACCGTTGTGTTATGCG
seq1-20	5815	54	0.735%	CACGCATAACTGGGCAGGCCTGCACACCCGCCGGGGGCATTCATGTGTGTTATGCG
seq1-21	5373	67	0.677%	CACGCATAACCATGTCTTGCAGATCATGGCCCCGCGGAATTCATGTGTGTTATGCG
seq1-22	5282	32	0.670%	CACGCATAACCGGCCCAATCGCATGCAGGTTCATCGTTGACCACAGGTGTTATGCG
seq1-23	5282	40	0.669%	CACGCATAACCGTTGCCATCGCATGGCAGCTACTGCACCACTCCCTGTGTTATGCG
seq1-24	5102	20	0.648%	CACGCATAACTCCACAAGCGCATGTGTGTATGCCCATTAAGGGATGGTGTTATGCG
seq1-25	5055	74	0.635%	CACGCATAACTTGTCATTGCATGACAGACGCTGGTGGCACTCGTGTGTGTTATGCG
seq1-26	4926	33	0.624%	CACGCATAACCGCGAATGGCAGTGGCGCCTTACCGGTGGATCTCTGGTGTTATGCG
seq1-27	4614	29	0.585%	CACGCATAACCGAATGTCGCACATTAGGCGCTGCGGCAACTCGTGTGTGTTATGCG
seq1-28	4494	90	0.562%	CACGCATAACGACCGTCGGATGACAGTTATGCTTCCTTGTGCCCCAGTGTTATGCG
seq1-29	4413	24	0.560%	CACGCATAACCATCCCACTGTTCGGATGCCGCTGGGGAATTTATGAGTGTTATGCG
seq1-30	4298	37	0.544%	CACGCATAACCCGTCGCTAAGCCGAAGTGTGTGTTTCCAGTCGTGGGTGTTATGCG

total reads: 783881

Table S3. Top 30 aptmers nanoflow result.

Aptmer name	positive vesicles proportion
seq1-01	1.30%
seq1-02	0.80%
seq1-03	0.30%
seq1-04	0.20%
seq1-05	0.30%
seq1-06	0.20%
seq1-07	0.60%
seq1-08	0.50%
seq1-09	0.70%
seq1-10	0.90%
seq1-11	0.50%
seq1-12	0.40%
seq1-13	0.50%
seq1-14	0.20%
seq1-15	0.50%
seq1-16	0.50%
seq1-17	0.20%
seq1-18	0.10%
seq1-19	0.20%
seq1-20	0.10%
seq1-21	0.20%
seq1-22	0.40%
seq1-23	0.50%
seq1-24	0,2%
seq1-25	**16.40%**
seq1-26	0.40%
seq1-27	0.30%
seq1-28	0.80%
seq1-29	0.30%
seq1-30	1.30%

Table S4. Library information.

Primer name	sequence (5' to 3')	Purpose
Lib2-76nt	GGGACCAGCACACGCATAACNNNNNNNNNNNNNNNNNNNNNNNNNNN NNNNNNNNNGTGTTATGCGTGCTACCGTG	initial library information
Lib1S1	GGGACCAGCACACGCATAAC	qPCR detection
Lib2A2	CACGGTAGCACGCATAACAC	
Lib2A2-ployA	AAAAAAAAAAAAAAAAAAAAAAAAA-Spacer18-CACGGTAGCACGCATAACAC	single chains preparation
Lib1S1-FAM	GGGACCAGCACACGCATAAC	
Lib1S1CS-Biotin	GTTATGCGTGTGCTGGTCCC-Biotin	fixed library

Table S5. Patient clinical information.

Sample number	Diagnosis	Age	Gleason score	Initial PSA
PCa1	PCa	70	7	63.54
PCa2	PCa	73	6	14.3
PCa3	PCa	74	8	65
PCa4	PCa	59	7	26.45
PCa5	PCa	71	9	4.5
PCa6	PCa	62	8	349.6
PCa7	PCa	69	9	21.28
PCa8	PCa	81	7	8.4
PCa9	PCa	67	9	37.21
PCa10	PCa	58	7	12.34
PCa21	PCa	73	7	26.23
BPH1	BPH	51	-	5.06
BPH2	BPH	58	-	1.24
BPH3	BPH	68	-	8.01
BPH4	BPH	63	-	2.75
BPH5	BPH	75	-	3.41
BPH6	BPH	80	-	2.1
BPH7	BPH	73	-	12.35
BPH8	BPH	77	-	0.92
BPH9	BPH	65	-	4.39
BPH10	BPH	68	-	2.53
