# Supplementary material for: Identification and diagnostic evaluation of an aptamer targeting prostate-cancer-derived small extracellular vesicles
Source: Mol Ther Nucleic Acids. 2026 Jan 15;37(1):102836. doi: 10.1016/j.omtn.2026.102836 (PMC12870759; doi:10.1016/j.omtn.2026.102836)
Supplement: Document S2. Article plus supplemental information [file mmc2.pdf]

Molecular Therapy
Nucleic Acids
Brief report

Identification and diagnostic evaluation of an aptamer targeting prostate-cancer-derived small extracellular vesicles

Ting Ding,[1,2,3,7] Yue Li,[1,7] Li Xue,[4,7] Chaoliang Xiong,[1] Lijuan Yu,[5] Qian He,[1] Jiayun Liu,[2] Xiaoke Hao,[2,3,6] and Dan Zhao[1,2]

[1]Department of Clinical Laboratory, The Second Affiliated Hospital of Xi'an Jiaotong University, Xi'an 710000, China; [2]Department of Clinical Laboratory, Xijing Hospital, Fourth Military Medical University (Air Force Medical University), Xi'an 710032, China; [3]School of Medicine, Northwest University, Xi'an 710069, China; [4]Department of Urology, The Second Affiliated Hospital of Xi'an Jiaotong University, Xi'an 710000, China; [5]Sahlgrenska Center for Cancer Research, Department of Oncology, Institute of Clinical Sciences, Sahlgreska Academy, University of Gothenburg, Gothenburg 40530, Sweden; [6]Xi'an Area Medical Laboratory Center, Xi'an 710100, China

Prostate cancer (PCa) lacks convenient, non-invasive, and highly specific diagnostic markers. Aptamers have emerged as preferred probes for biosensors that target extracellular vesicles (EVs). This study aimed to explore the diagnostic value of PCa-specific EVs aptamer probes. We used EV-SELEX to identify aptamers that selectively target PCa small EVs (sEVs). Surface plasmon resonance (SPR) and nanoflow cytometry were used to verify aptamer affinity. The diagnostic value of PCa was evaluated using clinical samples from patients. We screened and validated an aptamer, seq25, which exhibited high specificity for PCa-derived sEVs. The SPR assay revealed a strong binding affinity, with a KD of 24.02 nM and a dose-dependent binding response. Nanoflow cytometry demonstrated that seq25 could distinguish sEVs from PCa and normal prostate cell lines. In clinical specimens, the proportion of seq25-positive sEVs isolated from urine samples was significantly higher in patients with PCa than in those with benign prostatic hyperplasia. Our study integrated the diagnostic advantages of EVs with the technical benefits of aptamers to develop a PCa-specific sEVs aptamer probe that offers a promising non-invasive approach for PCa diagnosis.

INTRODUCTION

Prostate cancer (PCa) is one of the most prevalent malignancies in men and poses a significant global health burden.[1] Currently, prostate-specific antigen (PSA) is the most commonly used serum biomarker for PCa screening in high-risk populations due to its cost-effectiveness and accessibility.[2] However, the clinical application of PSA is limited by its inability to differentiate between benign prostatic hyperplasia and malignant lesions and to distinguish between low-risk and high-risk PCa.[3] Therefore, the development of novel PCa biomarkers is critical.

Liquid biopsy is an advanced technology for cancer detection that overcomes the challenges of difficult tissue sampling and tumor heterogeneity. Extracellular vesicles (EVs), one of the three core targets of liquid biopsy, along with circulating tumor DNA and cells, have gained widespread recognition for their diagnostic utility in oncology.[4] Nevertheless, the presence of abundant EVs derived from normal cells in body fluids considerably dilutes tumor-derived signals, making specific isolation and detection of tumor-derived EVs a crucial challenge.[5] Although studies have explored the application of EVs in PCa diagnosis,[6] most methods require complex preprocessing for EV and cargo extraction, which severely limits their practical application in clinical diagnostics. As functional nucleic acid molecules, aptamers are ideal candidates for constructing various biosensors due to their stability, binding affinity, ease of modification, and low cost.[7] This provides a potential solution for specific isolation and detection of tumor-derived EVs.

In this study, we explored the feasibility of using specific aptamers to identify PCa cell-derived small EVs (sEVs). By developing a novel differential-SELEX (EV-SELEX) screening method, we obtained aptamers that specifically target PCa-derived sEVs, which effectively distinguish sEVs of urine samples from patients with PCa and those with benign prostatic hyperplasia (BPH). This study highlights the potential of aptamers targeting specific EV subpopulations as novel tools for liquid biopsy in oncology, thereby providing an innovative approach for the clinical diagnosis of PCa.

Received 12 October 2025; accepted 12 January 2026;
https://doi.org/10.1016/j.omtn.2026.102836.

[7]These authors contributed equally

Correspondence: Jiayun Liu, Department of Clinical Laboratory, Xijing Hospital, Fourth Military Medical University (Air Force Medical University), Xi'an 710032, China.
E-mail: drjiayunliu@163.com
Correspondence: Xiaoke Hao, Department of Clinical Laboratory, The Second Affiliated Hospital of Xi'an Jiaotong University, Xi'an 710000, China.
E-mail: drhaoxk@163.com
Correspondence: Dan Zhao, Department of Clinical Laboratory, The Second Affiliated Hospital of Xi'an Jiaotong University, Xi'an 710000, China.
E-mail: zd2022xxyy@fmmu.edu.cn

© 2026 The Authors.
Published by Elsevier Inc. on behalf of The American Society of Gene and Cell Therapy.
This is an open access article under the CC BY license (http://creativecommons.org/licenses/by/4.0/).

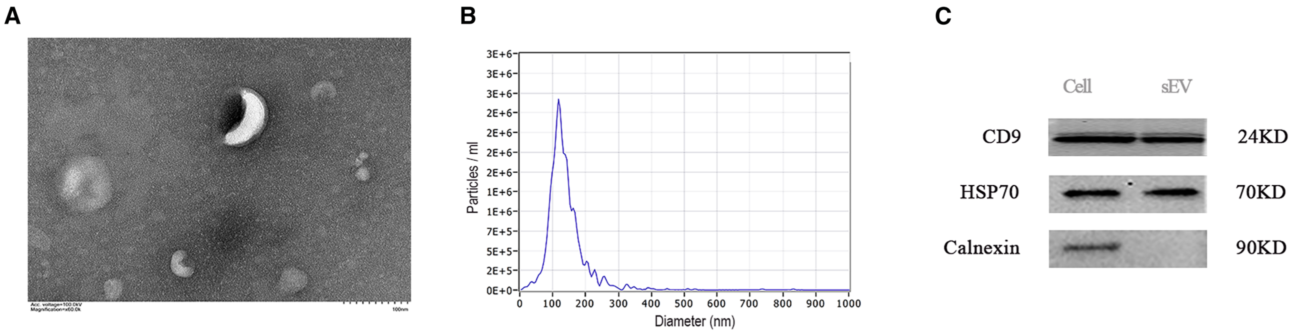

Figure 1. Isolation and characterization of sEVs

(A) Representative TEM images of sEVs. Scale bars, 100 nm. (B) NTA results showing size distribution of sEVs in the range 80–200 nm. (C) Relative expression levels of sEV protein markers (CD9, HSP70) and negative control (calnexin) by western blot.

RESULTS

Isolation and characterization of sEV

We extracted and characterized sEVs from the culture supernatants of prostate-derived cell lines. Transmission electron microscopy (TEM) revealed that the isolated sEVs exhibited classical cup-shaped morphology (Figure 1A), with an average size of approximately 120 nm (Figure 1B). Western blot analysis confirmed the presence of positive markers (CD9 and HSP70) and absence of a negative marker (calnexin) for EVs (Figure 1C).

Screening and identification of specific aptamers targeting PCa-derived sEVs

PCa sEV-specific aptamers were screened using the EV-SELEX method (Figure 2A). Nine screening rounds were performed. The screening conditions are listed in Table S1. With each SELEX round, aptamers bound to positively selected regions were progressively enriched (Figure 2B). We used fluorescence polarization (FP)[8] to detect library affinity in the selected rounds. These results suggest that the library affinity for positive selection gradually increased with each SELEX round (Figure 2C). Sequencing was performed on the library obtained in the final round. The sequences and enrichment rates of the top 30 aptamers are listed in Table S2. Next, surface plasmon resonance (SPR) was used to detect the affinities of the top 30 aptamers (Figure 2D). The affinity of seq25 for positive selection was significantly higher than that of the other aptamers, with a KD of 24.02 nM (Figure 2E). Given the limitations of SPR in clinical analysis, we used nanoflow cytometry as an alternative approach to validate the binding affinity. At the same concentration, only seq25 showed good binding to positively selected aptamers, with a high proportion of positive vesicles after incubation (Table S3). Its binding to negatively selected vesicles was negligible, with a low proportion of positive vesicles (Figure S1). Moreover, we compared seq25 with a well-characterized PCa-associated PSMA-specific aptamer (A10–3.2; Figure S1). Under the same experimental conditions, we observed that the performance of A10–3.2 was suboptimal, possibly because the PCa-cell-line-derived EV pool used in our positive selection included EVs from PSMA-negative lines (DU145 and PC3). These findings suggest that seq25 can achieve functional complementarity with existing PCa-related aptamers, which has significant implications for improving PCa diagnosis. We observed a dose-dependent interaction between seq25 and positive selection (Figure 2F). Seq25 at 100 nM enabled approximately 10% of the positively selected sEVs to exhibit fluorescence (concentration standardized to 1×10^8 particles/mL).

Potential value of selective fluorescent aptamers in PCa clinical diagnosis

The secondary structure of aptamer seq25 is shown in Figure 3A. To further explore the potential of seq25 in the diagnosis of PCa, we used flow cytometry to assess positive vesicle proportions in different samples after co-incubation with seq25. First, we analyzed cell-line-derived samples and observed that the proportion of positive vesicles in sEVs from PCa cell lines (DU145 and LNCaP) was significantly higher than that in sEVs from a normal immortalized prostate cell line (RWPE-1) (Figures 3B–3E). Subsequently, we extended our analysis to sEVs isolated from urine samples of 10 patients with PCa and 10 with BPH. The findings demonstrated that sEVs from patients with PCa showed a markedly higher proportion of positive vesicles following co-incubation with seq25 (Figure 3F), indicating their potential significance in the diagnosis of PCa. To quantify the diagnostic performance, receiver operating characteristic curve analysis was conducted, which yielded an area under the curve (AUC) of 0.945 (95% confidence interval [CI]: 0.85–1.00), with a sensitivity and specificity of 0.90 each at the optimal cutoff of 1.75 (Figure 3G).

DISCUSSION

PCa remains a significant global health burden,[1,9] with current diagnostic tools such as PSA testing demonstrating limited specificity in distinguishing malignant from benign conditions and inadequate capacity for disease stratification.[3] EVs have emerged as promising biomarkers for PCa due to their high abundance and accessibility in biofluids.[6,10] For instance, Bio-Techne's ExoDx Prostate IntelliScore employs an EV-derived RNA signature to enhance PCa diagnosis while reducing unnecessary biopsies.[11] Similarly, EV-associated proteins,[12] metabolites,[13,14] and DNA[15,16] are being actively investigated for their diagnostic potential, highlighting the growing potential of

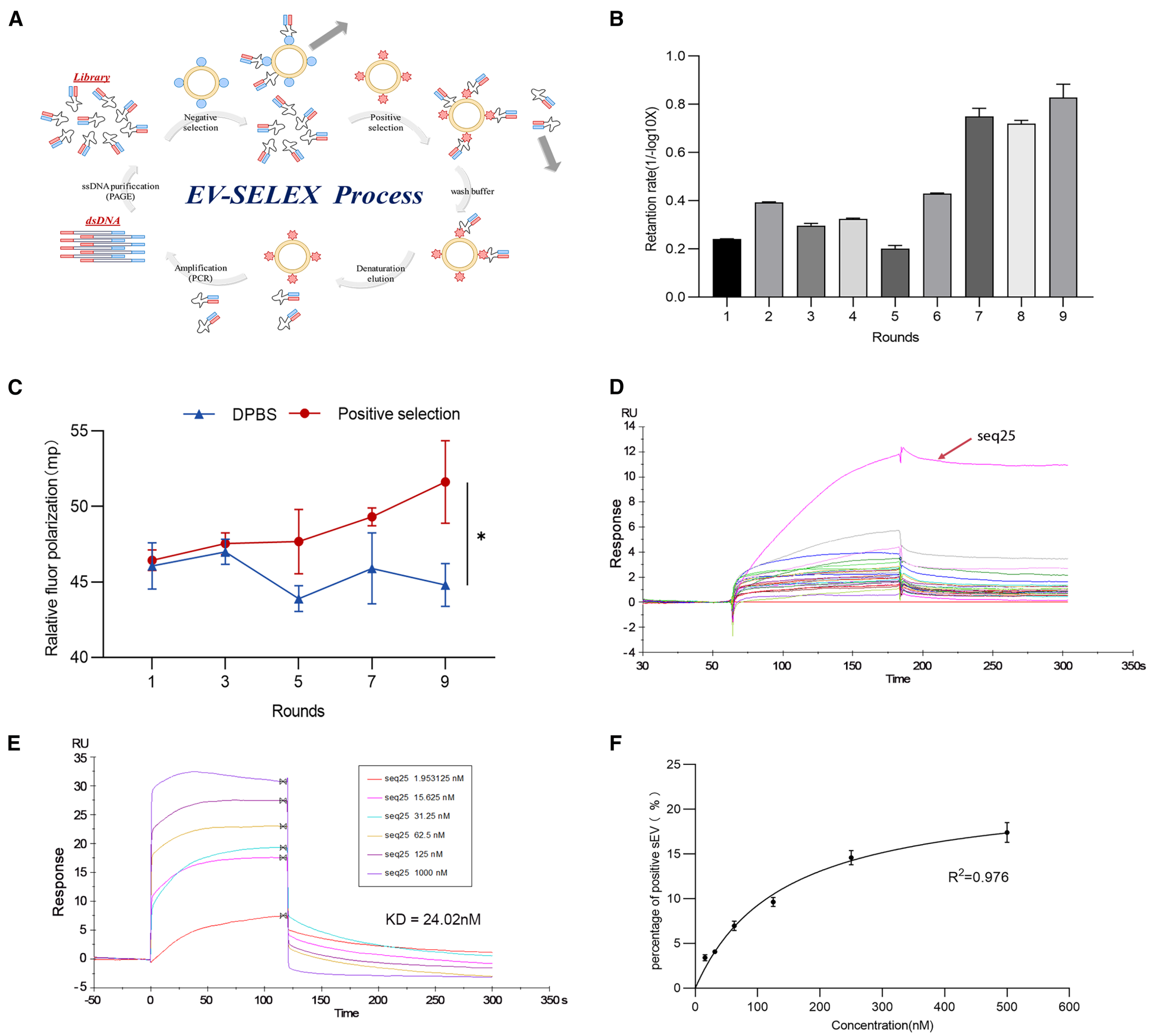

Figure 2. Screening and identification of specific aptamers targeting PCa-derived sEVs

(A) Schematic diagram of the process of PCa sEV-specific aptamers screening by EV-SELEX method. (B) Retention of positive selection binding aptamers in each round of EV-SELEX detected by PCR method. With the increase of SELEX rounds, aptamers bound to positive selection were gradually enriched. (C) The affinity of the library to positive target was detected by fluorescence polarization (FP). DPBS was used as a control. The *y* axis represents the fluorescence intensity. Higher values representing stronger affinity. With the increase of SELEX rounds, the affinity of the library for positive selection gradually increased. Data were analyzed using *t* test. $*p < 0.05$. (D) Affinity detection of top 30 aptamer with positive selection by surface plasmon resonance (SPR). The *y* axis represents the signal intensity. The affinity of seq25 for positive selection was significantly higher than other aptamers. (E) Binding affinity of aptamer seq25 to positive selection detected by SPR. The *y* axis represents the signal intensity. The curves in different colors represent the reactions of different concentrations of aptamers, with a KD of 24.02 nM. (F) Binding affinity of aptamer seq25 to positive selection detected by Nanoflow. The *y* axis represents the proportion of positive vesicles, and the *x* axis represents the different aptamer concentrations. There was a good dose-dependent interaction between seq25 and positive selection. The R^2 of the curve was 0.976.

EV-based liquid biopsies in PCa management. However, most current EV-based diagnostic studies analyze total EVs, which contain a substantial proportion of vesicles derived from normal cells. Isolating and detecting tumor-specific EVs could potentially enhance diagnostic sensitivity and specificity. For instance, Castillo et al. demonstrated that specifically isolating pancreatic-cancer-derived EVs improved the detection rate of KRAS mutations.[5] Similarly, Ju et al. showed that detecting the B7-H3 + EV subpopulation in prostate cancer patient plasma offered significant advantages in companion diagnostics for targeted therapy.[17] This study aims to

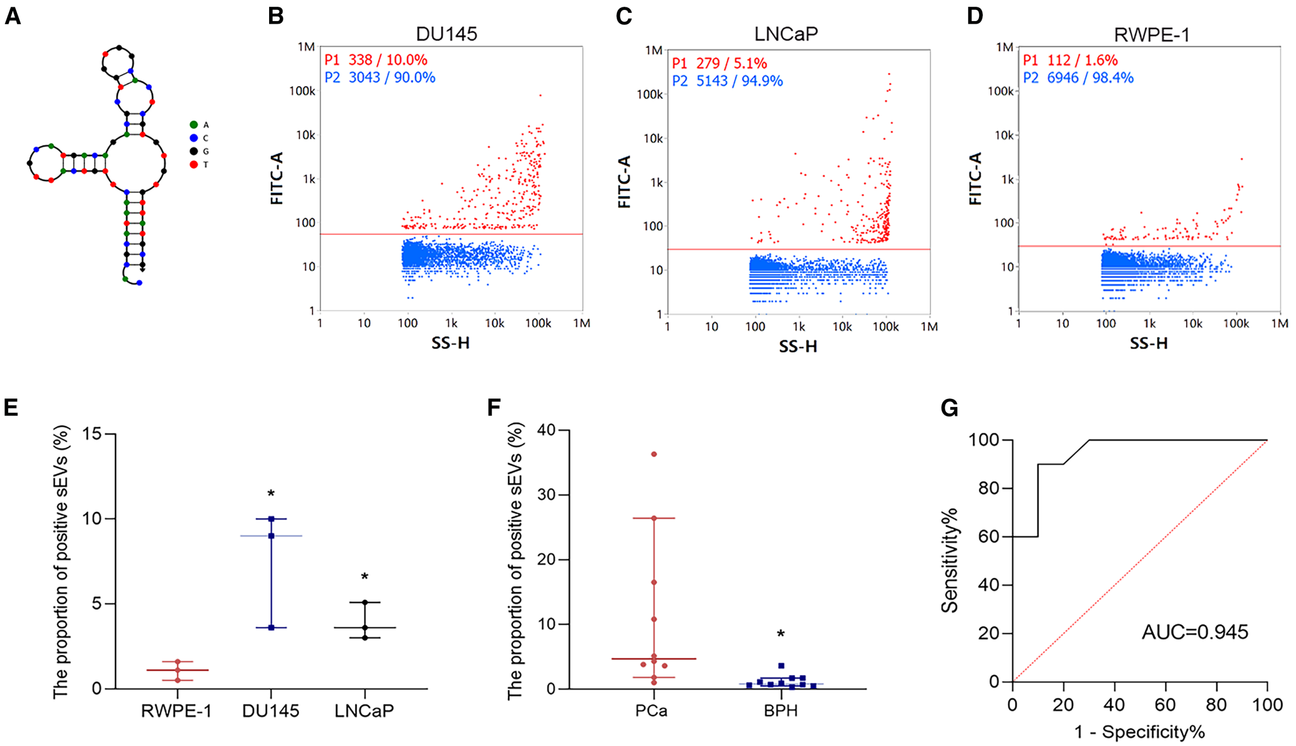

Figure 3. Potential value of selective fluorescent aptamers seq25 in PCa diagnosis

(A) The secondary structures of aptamers seq25. (B–D) Representative scatterplots of the positive vesicles proportion for sEVs from different prostate cell line after co-incubation with seq25 detected by Nanoflow, including two PCa cell lines (B, DU145; C, LNCaP) and a normal immortalized prostate cell lines (D, RWPE-1). (E) Comparison of the positive vesicles proportion of sEVs from different cell lines after co-incubation with seq25. (F) Comparison of the positive vesicles proportion between urine sEVs from PCa and BPH patients after co-incubation with seq25. (G) ROC curve of seq25-positive vesicle proportion for distinguishing PCa from BPH. Data were analyzed using *t* test. **p* < 0.05.

identify a PCa EV-specific aptamer, which will hold great significance for the future isolation and detection of PCa-specific EVs.

Aptamers have gained considerable attention in diagnostic applications owing to their low immunogenicity, chemical modifiability, and cost-effectiveness.[7] Recent advances highlight their significant potential for EV detection and isolation, including highly sensitive quantitative detection, precise subtype discrimination, and functional modulation.[18] For quantitative analysis, advanced platforms such as artificial-nucleotide-aptamer-based field-effect transistors (AN-Apta-FET) achieve ultra-low detection limits (242 particles/mL) for hepatoma EVs, distinguishing clinical serum samples within 9 min,[19] while ExoAPP (exosome-oriented, aptamer nanoprobe-enabled profiling) integrates graphene oxide, target-responsive aptamers, and enzyme-assisted signal amplification to reach a detection limit of 1.6×10^5 particles/mL.[20] For subtype discrimination, Hornung et al. identified an aptamer distinguishing PCa subtypes (VCaP vs. LNCaP).[21] In contrast to aptamers distinguishing PCa subtypes, our work focused on developing a pan-PCA sEV-targeting aptamer to discriminate malignancy from benign conditions. Functionally, aptamers such as ex-50.T extend beyond detection to inhibit EV uptake and antagonize cancer-EV-induced cell migration *in vitro*,[22] offering therapeutic potential that our current study (focused on diagnostic utility) does not yet explore. These developments underscore the unique capability of aptamer technology in precise EV targeting, potentially enabling enhanced diagnostic accuracy and therapeutic interventions.

In this study, we identified a novel aptamer (seq25) demonstrating specific binding affinity for PCa-derived sEVs. Our findings reveal that seq25 effectively discriminates between sEVs from PCa patients and those with benign prostatic hyperplasia, suggesting its utility as a non-invasive diagnostic tool. However, several limitations should be acknowledged: the sample size was relatively small and requires larger-scale validation. The detection still depends on EV extraction; future work should aim to develop extraction-free protocols to enhance clinical feasibility. Furthermore, both the sensitivity/specificity of the assay and the target of the aptamer need further optimization and clarification to solidify the mechanistic basis. Notwithstanding these limitations, our work provides a proof of concept for using aptamer-based probes to target disease-specific sEVs, establishing a foundation for the development of novel non-invasive diagnostic and future theranostic tools for PCa.

MATERIALS AND METHODS

Cell culture

We used three PCa cell lines (DU145, PC3, and LNCaP) and two normal immortalized prostate cell lines (RWPE-1 and WPMY-1). DU145 and WPMY-1 cells were maintained in high-glucose Dulbecco's modified Eagle's medium containing 10% fetal bovine serum (FBS). PC3 cells were maintained in Ham's F-12K medium containing 10% FBS. LNCaP cells were maintained in RPMI-1640 medium containing 10% FBS. RWPE-1 cells were maintained in keratinocyte medium containing 1% keratinocyte growth factor. The added FBS was replaced with exosome-depleted FBS before EV extraction.

sEVs isolation and characterization

Cell culture supernatants and urine (50 mL samples of freshly retained first-voided urine) were extracted using ultracentrifugation as previously described.[23] The specific details can be found in the Supplementary Material. The extracted sEVs were identified according to the guidelines of ISEV.[24] First, representative sEV morphologies were captured by TEM (Tecnai, USA). Then, the size distribution and concentration of sEVs were measured using nanoparticle tracking analysis (NTA) with the ZetaView instrument (Particle Metrix, Germany). Finally, as described earlier,[15] the extracted sEV samples were detected by western blot (WB) using two EV positive markers and one EV negative marker: CD9 (Abcam ab20597, 1:3,000), HSP70 (Abcam ab18169, 1:1,000), and Calnexin (Abcam ab133615, 1:1,000).

EV-SELEX

We used EV-SELEX method (Figure 2A) to screen PCa sEV-specific aptamers with reference to previous reports with some modifications.[21] The specific details can be found in the Supplementary Material. SEVs isolated from PCa cell lines were employed for positive selection, while those derived from normal immortalized prostate cell lines served as negative selection. Library information is provided in Table S4. A total of nine rounds of screening were conducted. The specific screening conditions were shown in Table S1.

Library affinity assay

The affinity of the library to positive selection was detected by fluorescence polarization (FP). Libraries obtained in 1, 3, 5, 7, and 9 rounds were diluted to 100 nM with DPBS, at 95°C for 10 min followed by an immediate ice water bath for 5 min. Thirty microliters of each sample was incubated with 5 μL of 0.5 μg/μL positive selection reagent for 30 min at room temperature and subsequently detected by Varioskan LUX (Thermo Fisher Scientific).

Sequence analysis

The library from the final round of SELEX was sent to Sangon Biotechnology (Shanghai, China) for high-throughput sequencing by an Illumina high-throughput sequencing platform. The results of aptamers sequencing were analyzed by DNAMAN software. The most frequent and enriched aptamer sequences were chosen for further analysis. The secondary structure of aptamers was analyzed using NUPACK (https://nupack.org/). The enrichment rate of the aptamer was calculated based on the proportion of reads obtained from sequencing. The sequence and the enrichment rate results of the top 30 aptamers were shown in Table S2.

Surface plasmon resonance

Surface plasmon resonance (SPR) was used to detect the affinity of the top 30 aptamers ranked by enrichment rate, and the aptamer with the highest affinity was selected for KD value determination. The positive selection sample was diluted to a final concentration of 50 μg/mL using 10 mM sodium acetate buffer (pH 4.0) and injected at a flow rate of 5 μL/min for 600 s to immobilize it on a CM5 chip (GE Healthcare, USA). The chip was subsequently blocked with ethanolamine solution at the same flow rate of 5 μL/min for 10 min. The target aptamer was then diluted to the desired concentration in PBST buffer and analyzed using an SPR instrument (GE Healthcare, USA) with an injection contact time of 120 s.

Nanoflow cytometry

Nanoflow cytometry was employed to further assess the affinity of the aptamers for positive selection. The top 30 aptamers, A10–3.2 (PSMA apatmer, RiboBio, Guangzhou, China), and seq25 (all 5′-FAM-labeled) at different concentrations were incubated at 95°C for 10 min, followed by rapid cooling in an ice-water bath for 5 min. The sEV samples were adjusted to a concentration of 1×10^8 particles/mL and co-incubated with aptamers at room temperature in the dark for 30 min. Free dye was removed by centrifuging the stained sEV samples using a 0.5 mL 100 kDa ultrafiltration tube (Millipore, USA). The proportion of aptamer-positive vesicles was then quantified by nanoflow cytometry.[25] The instrument settings and gating strategy are detailed in the Supplementary Material. Final validation of seq25 performance was performed using cell-derived and clinical urine-derived sEV samples.

Patients

We recruited 10 treatment-naïve PCa patients and 10 BPH patients confirmed by histopathology at the First Affiliated Hospital of the Air Force Medical University and collected baseline clinical information and urine samples. The research was conducted with the approval of the hospital's ethics committee (NO.KY20222066-C-1), and patient samples were collected following the acquisition of informed consent. Detailed clinical information regarding the patients is provided in Table S5.

Statistical analysis

Data analysis was performed using GraphPad Prism software (v.9.5.1). Statistical comparisons were conducted utilizing t tests. All tests were two tailed, and a p value of less than 0.05 was deemed statistically significant (n.s. no significance, $^{*}p < 0.05$; $^{**}p < 0.01$; $^{***}p < 0.001$).

DATA AND CODE AVAILABILITY

The data presented in this study are available on request from the corresponding author.

ACKNOWLEDGMENTS

This study was conducted in accordance with the principles of the Declaration of Helsinki and approved by the ethics committee of the Xijing Hospital affiliated to Air Force Medical University (no. KY20222066-C-1). The samples and clinical information were obtained with the patient's written informed consent. This work was supported by the National Natural Science Foundation of China (no. 8217102695), the Scientists and Engineers Team Construction Project of Shaanxi Province (no. 2022KXJ-103), and the Shaanxi Provincial Natural Science Basic Research Program (no. 2025JC-YBQN-1185).

AUTHOR CONTRIBUTIONS

Conceptualization, T.D., J.L., X.H., and D.Z.; data curation, T.D., Y.L., L.X., C.X., and L.Y.; methodology, T.D., Q.H., and D.Z.; investigation, T.D., Y.L., L.X., C.X., J.L., and X.H.; writing—original draft preparation, T.D. and D.Z.; writing—review and editing, Y.L., L.X., C.X., L.Y., Q.H., J.L., and X.H. All authors have read and agreed to the published version of the manuscript.

DECLARATION OF INTERESTS

The authors declare no conflict of interest.

SUPPLEMENTAL INFORMATION

Supplemental information can be found online at https://doi.org/10.1016/j.omtn.2026.102836.

REFERENCES

1. Bergengren, O., Pekala, K.R., Matsoukas, K., Fainberg, J., Mungovan, S.F., Bratt, O., Bray, F., Brawley, O., Luckenbaugh, A.N., Mucci, L., et al. (2023). 2022 Update on Prostate Cancer Epidemiology and Risk Factors-A Systematic Review. Eur. Urol. *84*, 191–206. https://doi.org/10.1016/j.eururo.2023.04.021.

2. Adamaki, M., and Zoumpourlis, V. (2021). Prostate Cancer Biomarkers: From diagnosis to prognosis and precision-guided therapeutics. Pharmacol. Ther. *228*, 107932. https://doi.org/10.1016/j.pharmthera.2021.107932.

3. Louie, K.S., Seigneurin, A., Cathcart, P., and Sasieni, P. (2015). Do prostate cancer risk models improve the predictive accuracy of PSA screening? A meta-analysis. Ann. Oncol. *26*, 848–864. https://doi.org/10.1093/annonc/mdu525.

4. Heitzer, E., Haque, I.S., Roberts, C.E.S., and Speicher, M.R. (2019). Current and future perspectives of liquid biopsies in genomics-driven oncology. Nat. Rev. Genet. *20*, 71–88. https://doi.org/10.1038/s41576-018-0071-5.

5. Castillo, J., Bernard, V., San Lucas, F.A., Allenson, K., Capello, M., Kim, D.U., Gascoyne, P., Mulu, F.C., Stephens, B.M., Huang, J., et al. (2018). Surfaceome profiling enables isolation of cancer-specific exosomal cargo in liquid biopsies from pancreatic cancer patients. Ann. Oncol. *29*, 223–229. https://doi.org/10.1093/annonc/mdx542.

6. Robinson, H., Roberts, M.J., Gardiner, R.A., and Hill, M.M. (2023). Extracellular vesicles for precision medicine in prostate cancer - Is it ready for clinical translation? Semin. Cancer Biol. *89*, 18–29. https://doi.org/10.1016/j.semcancer.2023.01.003.

7. Rozenblum, G.T., Lopez, V.G., Vitullo, A.D., and Radrizzani, M. (2016). Aptamers: current challenges and future prospects. Expert Opin. Drug Discov. *11*, 127–135. https://doi.org/10.1517/17460441.2016.1126244.

8. Zhang, Z., Tang, C., Zhao, L., Xu, L., Zhou, W., Dong, Z., Yang, Y., Xie, Q., and Fang, X. (2019). Aptamer-based fluorescence polarization assay for separation-free exosome quantification. Nanoscale *11*, 10106–10113. https://doi.org/10.1039/c9nr01589b.

9. Liu, J., Dong, L., Zhu, Y., Dong, B., Sha, J., Zhu, H.H., Pan, J., and Xue, W. (2022). Prostate cancer treatment – China's perspective. Cancer Lett. *550*, 215927. https://doi.org/10.1016/j.canlet.2022.215927.

10. Pang, B., Zhu, Y., Ni, J., Thompson, J., Malouf, D., Bucci, J., Graham, P., and Li, Y. (2020). Extracellular vesicles: the next generation of biomarkers for liquid biopsy-based prostate cancer diagnosis. Theranostics *10*, 2309–2326. https://doi.org/10.7150/thno.39486.

11. McKiernan, J., Donovan, M.J., O'Neill, V., Bentink, S., Noerholm, M., Belzer, S., Skog, J., Kattan, M.W., Partin, A., Andriole, G., et al. (2016). A Novel Urine Exosome Gene Expression Assay to Predict High-grade Prostate Cancer at Initial Biopsy. JAMA Oncol. *2*, 882–889. https://doi.org/10.1001/jamaoncol.2016.0097.

12. Bernardino, R.M.M., Leao, R., Henrique, R., Pinheiro, L.C., Kumar, P., Suravajhala, P., Beck, H.C., Carvalho, A.S., and Matthiesen, R. (2021). Extracellular Vesicle Proteome in Prostate Cancer: A Comparative Analysis of Mass Spectrometry Studies. Int. J. Mol. Sci. *22*. https://doi.org/10.3390/ijms222413605.

13. Ding, T., He, W., Yan, H., Wei, Z., Zeng, X., and Hao, X. (2024). Metabolic profiling in tissues and urine of patients with prostatic lesions and the diagnostic value of urine extracellular vesicles metabolites in prostate cancer. Clin. Chim. Acta *556*, 117845. https://doi.org/10.1016/j.cca.2024.117845.

14. Hamed, M.A., Wasinger, V., Wang, Q., Graham, P., Malouf, D., Bucci, J., and Li, Y. (2024). Prostate cancer-derived extracellular vesicles metabolic biomarkers: Emerging roles for diagnosis and prognosis. J. Control. Release *371*, 126–145. https://doi.org/10.1016/j.jconrel.2024.05.029.

15. Ding, T., Diao, Y., Fu, R., Gong, C., He, Q., He, W., Zhang, L., Yang, X., Zeng, X., Yu, L., et al. (2025). The significance of urine extracellular vesicle DNA methylation detection in the diagnosis and classification of prostate cancer. J. Adv. Res. https://doi.org/10.1016/j.jare.2025.09.056.

16. Vagner, T., Spinelli, C., Minciacchi, V.R., Balaj, L., Zandian, M., Conley, A., Zijlstra, A., Freeman, M.R., Demichelis, F., De, S., et al. (2018). Large extracellular vesicles carry most of the tumour DNA circulating in prostate cancer patient plasma. J. Extracell. Vesicles *7*, 1505403. https://doi.org/10.1080/20013078.2018.1505403.

17. Ju, Y., Watson, J., Wang, J.J., Yen, Y.T., Gevorkian, L., Chen, Z., Tu, K.H., Salumbides, B., Phung, A., Zhao, C., et al. (2025). B7-H3-liquid biopsy for the characterization and monitoring of the dynamic biology of prostate cancer. Drug Resist. Updat. *79*, 101207. https://doi.org/10.1016/j.drup.2025.101207.

18. Zhu, C., Li, L., Wang, Z., Irfan, M., and Qu, F. (2020). Recent advances of aptasensors for exosomes detection. Biosens. Bioelectron. *160*, 112213. https://doi.org/10.1016/j.bios.2020.112213.

19. Chen, Y., Kong, D., Qiu, L., Wu, Y., Dai, C., Luo, S., Huang, Z., Lin, Q., Chen, H., Xie, S., et al. (2023). Artificial Nucleotide Aptamer-Based Field-Effect Transistor for Ultrasensitive Detection of Hepatoma Exosomes. Anal. Chem. *95*, 1446–1453. https://doi.org/10.1021/acs.analchem.2c04433.

20. Jin, D., Yang, F., Zhang, Y., Liu, L., Zhou, Y., Wang, F., and Zhang, G.J. (2018). ExoAPP: Exosome-Oriented, Aptamer Nanoprobe-Enabled Surface Proteins Profiling and Detection. Anal. Chem. *90*, 14402–14411. https://doi.org/10.1021/acs.analchem.8b03959.

21. Spetzler, D., Famulok, M., Mayer, G., Miglarese, M.R., Wei, X., Richards, M.N., Zhong, Z., Zarkovic, J., Maher, V., Tinder, T., et al. (2020). ADAPT identifies an ESCRT complex composition that discriminates VCaP from LNCaP prostate cancer cell exosomes. Nucleic Acids Res. *48*, 4013–4027. https://doi.org/10.1093/nar/gkaa034.

22. Esposito, C.L., Quintavalle, C., Ingenito, F., Rotoli, D., Roscigno, G., Nuzzo, S., Thomas, R., Catuogno, S., de Franciscis, V., and Condorelli, G. (2021). Identification of a novel RNA aptamer that selectively targets breast cancer exosomes. Mol. Ther. Nucleic Acids *23*, 982–994. https://doi.org/10.1016/j.omtn.2021.01.012.

23. Yu, L., Sui, B., Fan, W., Lei, L., Zhou, L., Yang, L., Diao, Y., Zhang, Y., Li, Z., Liu, J., and Hao, X. (2021). Exosomes derived from osteogenic tumor activate osteoclast differentiation and concurrently inhibit osteogenesis by transferring COL1A1-targeting miRNA-92a-1-5p. J. Extracell. Vesicles *10*, e12056. https://doi.org/10.1002/jev2.12056.

24. Welsh, J.A., Goberdhan, D.C.I., O'Driscoll, L., Buzas, E.I., Blenkiron, C., Bussolati, B., Cai, H., Di Vizio, D., Driedonks, T.A.P., Erdbrügger, U., et al. (2024). Minimal information for studies of extracellular vesicles (MISEV2023): From basic to advanced approaches. J. Extracell. Vesicles *13*, e12404. https://doi.org/10.1002/jev2.12404.

25. Liu, H., Tian, Y., Xue, C., Niu, Q., Chen, C., and Yan, X. (2022). Analysis of extracellular vesicle DNA at the single-vesicle level by nano-flow cytometry. J. Extracell. Vesicles *11*, e12206. https://doi.org/10.1002/jev2.12206.

OMTN, Volume *37*

Supplemental information

Identification and diagnostic evaluation of an aptamer targeting prostate-cancer-derived small extracellular vesicles

Ting Ding, Yue Li, Li Xue, Chaoliang Xiong, Lijuan Yu, Qian He, Jiayun Liu, Xiaoke Hao, and Dan Zhao

Supplemental materials and methods

sEVs isolation and characterization

The freshly collected samples were sequentially centrifuged at 300 g for 10 minutes to remove cells, at 2000 g for 20 minutes to eliminate cell debris, at 10000 g for 30 minutes to isolate large extracellular vesicles (lEVs), and finally at 120,000 g (Optima XPN-90, Type 45 Ti rotor, Beckman Coulter, USA) for 2.5 hours to isolate small extracellular vesicles (sEVs). Post-ultracentrifugation, the pellet was resuspended in PBS and stored at -80°C until use. sEVs morphology was analysed using transmission electron microscopy (TEM) (Tecnai, USA). In brief, vesicles were loaded onto the glow-discharged 300-mesh copper grids (Electron Microscopy Sciences, Hatfield, PA) for 30 seconds, then washed by water twice, and further stained with 2% uranyl formate for 1 min. Negative stained sEVs were analyzed by digitization on a FEI Tecnai G2 F20 X-TWIN Transmission Electron Microscope at 120 kV. To examine sEVs size distribution and particle concentration, nanoparticle tracking analysis (NTA) was performed using a ZetaView instrument (Particle Metrix, Germany) with a dilution of 1:10000, and parameters as below: sensitivity:80%, Max Area: 1000, Min Bright 30, Min Area 10.

EV-SELEX

SEVs isolated from PCa cell lines were employed for positive selection, while those derived from normal immortalized prostate cell lines served as negative selection. An ssDNA 76 nt randomized oligonucleotide library and required primers were chemically synthesized by Sangon Biotech (Shanghai, China) and the library information was provided in Table S4. To maintain EV membrane integrity and aptamer active conformation (and provide a suitable physicochemical environment for their specific binding), the binding reaction between EVs and aptamers was performed in the following system: reaction buffer (1× PBS, 3 mM $MgCl_2$, 0.5% Pluronic® F127). A total of nine rounds of screening were conducted (detailed conditions in Table S1), the key steps were as follows: The DNA library was subjected to denaturation (95°C for 10 min) and renaturation (ice bath for 5 min). For each round, the renatured library was first incubated with negative selection EVs for 1 hour. Aptamers bound to the negative

selection EVs were removed by centrifugation following the addition of PEG8000. The supernatant was collected and then incubated with positive selection EVs for 1 hour. Subsequently, the EV-aptamer complexes were precipitated with PEG8000, and the aptamers associated with positive selection EVs in the pellet were eluted with ultrapure water by boiling water bath. The eluates were quantified by Q-PCR, and the positive eluate was amplified by emulsion PCR (ePCR). Single-stranded DNA was isolated by denaturing PAGE electrophoresis, followed by dialysis, concentration, and quantification using a microvolume UV spectrophotometer. The enriched library was then used as the template for the next round of selection.

Nanoflow cytometry

Nanoflow cytometry analysis was performed using a Flow NanoAnalyzer (NanoFCM, China). The gating strategy to identify aptamer-positive EVs was as follows. Filtered phosphate-buffered saline (PBS) was used to establish the background scatter signal and exclude non-EV particles. Unstained EVs (not incubated with fluorescent aptamers) served as the negative control to define the autofluorescence threshold for the FAM channel. Events exceeding these thresholds were considered specifically stained EV populations. For signal detection, the light emitted by individual nanoparticles or EVs was collected perpendicularly to both the laser beam and the sample stream by an infinity-corrected microscope objective. Two single-photon counting avalanche photodiodes (APDs) were used for the simultaneous detection of side scatter and green FL (Fam signal) of individual particles, respectively. The 488 nm laser power was fixed at 20mW and the SS decay at 10%. The threshold levels for both the peak height (a digital discriminator level set to 3 times the standard deviation of the background) and the peak width of 0.2 and 0.3 ms were set as the criteria for burst (or peak) identification of SSC and FL signal, respectively. For each burst that satisfied the criteria, the integrated number of photons (background subtracted) was stored as the burst area for the histogram or dot-plot construction. The sampling pressure was fixed at 1.0 kPa prior to acquisition and events were recorded for a duration of 1 min for each sample.

Supplemental Figures

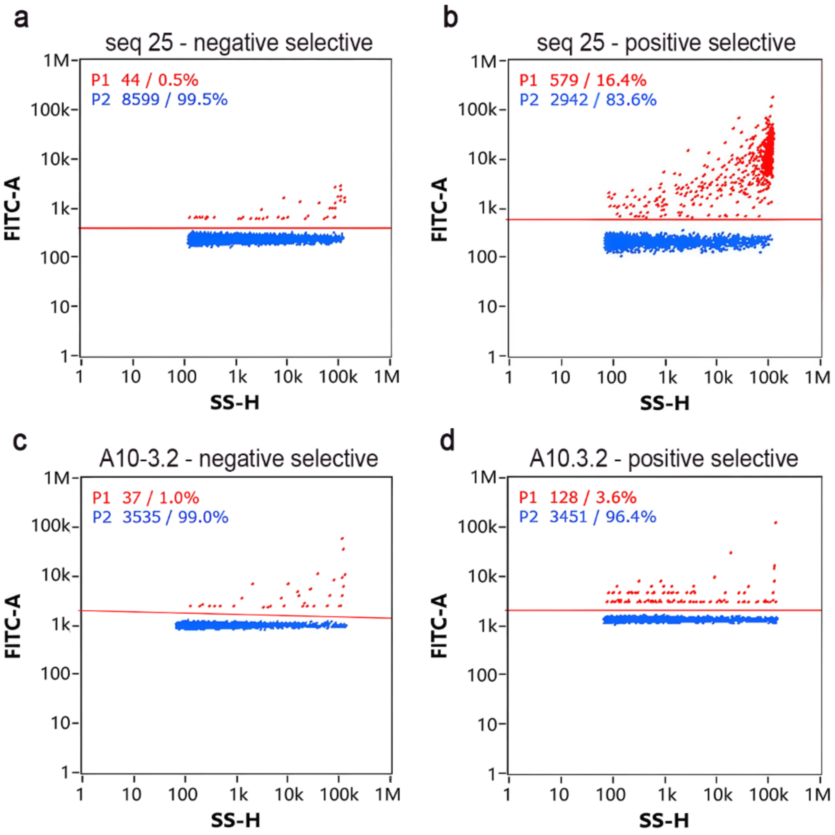

Figure S1. Comparison of binding specificity to PCa-EVs between seq25 and PSMA aptamer A10-3.2. a. Positive vesicles proportion of positive selection after co-incubation with seq 25 by Nanoflow. **b.** Positive vesicles proportion of negative selection after co-incubation with seq 25 by Nanoflow. **c.** Positive vesicles proportion of positive selection after co-incubation with A10-3.2 by Nanoflow. **d.** Positive vesicles proportion of negative selection after co-incubation with A10-3.2 by Nanoflow.

Table S1. Condition of EV-SELEX.

Round	Library	Negative selection	Positive selection
1	10μM, 137μL	0.6μg/μL, 50μl, 60min	0.5μg/μL, 60μl, 60min
2	700nM,150μL	0.6μg/μL, 50μl, 50min	0.5μg/μL, 60μl, 50min
3	560nM,100μL	0.6μg/μL, 50μl, 40min	0.5μg/μL, 60μl, 40min
4	400nM,150μL	0.6μg/μL, 50μl, 40min	0.5μg/μL, 60μl, 40min
5	300nM, 200μL	0.6μg/μL, 50μl, 30min	0.5μg/μL, 60μl, 30min
6	600nM,100μL	0.6μg/μL, 50μl, 30min	0.5μg/μL, 60μl, 30min
7	300nM,100μL+5ul, 1mg/ml salmon sperm DNA	0.6μg/μL, 50μl, 30min	0.5μg/μL, 60μl, 30min
8	300nM,100μL+10ul, 1mg/ml salmon sperm DNA	0.6μg/μL, 50μl, 30min	0.5μg/μL, 30μl, 30min
9	300nM,100μL+20ul, 1mg/ml salmon sperm DNA	0.6μg/μL, 50μl, 30min	0.5μg/μL, 30μl, 15min

Table S2. Sequencing results of the top 30 aptmers.

Aptmer name	Reads	Aerosol pollution	Enrichment ratio	Sequence
seq1-01	18726	68	2.380%	CACGCATAACTATCCACACCCGACCTCCGATCATGGCAGTTGCACTGTGTTATGCG
seq1-02	18230	54	2.319%	CACGCATAACTGTTCATGACACGTCACACTGCACCATCGATCGCATCGTGTTATGCG
seq1-03	16714	56	2.125%	CACGCATAACTCGACAACTGTCCGTCGACCGCTGGGGCATTCATGAGTGTTATGCG
seq1-04	16268	112	2.061%	CACGCATAACGCTCCATAGCATGGATCGCGCTGCCGGCACTTGTGTGTGTTATGCG
seq1-05	13545	39	1.723%	CACGCATAACCAAGCCGTAGTGTAGCGTAGTGTGTGTTTGCCCATGGTGTTATGCG
seq1-06	10370	158	1.303%	CACGCATAACGGGCCGGCTGTCGGATCCGTGCCGCGGAATTCATGTGTGTTATGCG
seq1-07	9576	80	1.211%	CACGCATAACGTCTCGCTCGCAGCGGACCCCTATCGGTGGCTCATTGGTGTTATGCG
seq1-08	9224	76	1.167%	CACGCATAACGAAGCATGCTAGGGATTCGCGCCGCGGCATTCATGTGTGTTATGCG
seq1-09	7997	47	1.014%	CACGCATAACCACCGACATCGCATGTCCACTTCAGTCGTCCTCGAGTGTTATGCG
seq1-10	7646	66	0.967%	CACGCATAACCGGTACAGCCAGGTAACGGCGCCGCGGCATTCATGTGTGTTATGCG
seq1-11	7409	44	0.940%	CACGCATAACTCCGCACAATTGCCTATGTGTTTGCCACCGGATGCAGTGTTATGCG
seq1-12	6527	41	0.827%	CACGCATAACGGACACCGTTCAAGGCCCTAGACAGCTACCTTTGAAGTGTTATGCG
seq1-13	6499	62	0.821%	CACGCATAACCGTGCATGGCATGTGCGCGGCCCGAGGCATTGATGTGTGTTATGCG
seq1-14	6252	76	0.788%	CACGCATAACCATGGGCCCGTCCCCGGCGAACAGATCGCATCTGGTGTGTTATGCG
seq1-15	6157	31	0.781%	CACGCATAACGACACCGTTGAACTGCTTCCACGAGCTTCCATTCAAGTGTTATGCG
seq1-16	6091	40	0.772%	CACGCATAACTGAGCACCTGTGAGCTCAACGCTGTGGCACTTGTCAGTGTTATGCG
seq1-17	6079	97	0.763%	CACGCATAACTGTCGGTTGCACAGACACTGGCATGGCCACTTGTAGGTGTTATGCG
seq1-18	5992	86	0.753%	CACGCATAACCCGGTCTCGCAGATCGGACGTTGTGACAGTTCTTGTGTGTTATGCG
seq1-19	5837	46	0.739%	CACGCATAACTGGCTAGATCGCATCGCAGTTCATCCCTGGACCGTTGTGTTATGCG
seq1-20	5815	54	0.735%	CACGCATAACTGGGCAGGCCTGCACACCCGCCGGGGGCATTCATGTGTGTTATGCG
seq1-21	5373	67	0.677%	CACGCATAACCATGTCTTGCAGATCATGGCCCCGCGGAATTCATGTGTGTTATGCG
seq1-22	5282	32	0.670%	CACGCATAACCGGCCCAATCGCATGCAGGTTCATCGTTGACCACAGGTGTTATGCG
seq1-23	5282	40	0.669%	CACGCATAACCGTTGCCATCGCATGGCAGCTACTGCACCACTCCCTGTGTTATGCG
seq1-24	5102	20	0.648%	CACGCATAACTCCACAAGCGCATGTGTGTATGCCCATTAAGGGATGGTGTTATGCG
seq1-25	5055	74	0.635%	CACGCATAACTTGTCATTGCATGACAGACGCTGGTGGCACTCGTGTGTGTTATGCG
seq1-26	4926	33	0.624%	CACGCATAACCGCGAATGGCAGTGGCGCCTTACCGGTGGATCTCTGGTGTTATGCG
seq1-27	4614	29	0.585%	CACGCATAACCGAATGTCGCACATTAGGCGCTGCGGCAACTCGTGTGTGTTATGCG
seq1-28	4494	90	0.562%	CACGCATAACGACCGTCGGATGACAGTTATGCTTCCTTGTGCCCCAGTGTTATGCG
seq1-29	4413	24	0.560%	CACGCATAACCATCCCACTGTTCGGATGCCGCTGGGGAATTTATGAGTGTTATGCG
seq1-30	4298	37	0.544%	CACGCATAACCCGTCGCTAAGCCGAAGTGTGTGTTTCCAGTCGTGGGTGTTATGCG

total reads: 783881

Table S3. Top 30 aptmers nanoflow result.

Aptmer name	positive vesicles proportion
seq1-01	1.30%
seq1-02	0.80%
seq1-03	0.30%
seq1-04	0.20%
seq1-05	0.30%
seq1-06	0.20%
seq1-07	0.60%
seq1-08	0.50%
seq1-09	0.70%
seq1-10	0.90%
seq1-11	0.50%
seq1-12	0.40%
seq1-13	0.50%
seq1-14	0.20%
seq1-15	0.50%
seq1-16	0.50%
seq1-17	0.20%
seq1-18	0.10%
seq1-19	0.20%
seq1-20	0.10%
seq1-21	0.20%
seq1-22	0.40%
seq1-23	0.50%
seq1-24	0,2%
seq1-25	**16.40%**
seq1-26	0.40%
seq1-27	0.30%
seq1-28	0.80%
seq1-29	0.30%
seq1-30	1.30%

Table S4. Library information.

Primer name	sequence (5' to 3')	Purpose
Lib2-76nt	GGGACCAGCACACGCATAACNNNNNNNNNNNNNNNNNNNNNNNNNNN NNNNNNNNNGTGTTATGCGTGCTACCGTG	initial library information
Lib1S1	GGGACCAGCACACGCATAAC	qPCR detection
Lib2A2	CACGGTAGCACGCATAACAC	qPCR detection
Lib2A2-ployA	AAAAAAAAAAAAAAAAAAAAAAAAA-Spacer18-CACGGTAGCACGCATAACAC	single chains preparation
Lib1S1-FAM	GGGACCAGCACACGCATAAC	single chains preparation
Lib1S1CS-Biotin	GTTATGCGTGTGCTGGTCCC-Biotin	fixed library

Table S5. Patient clinical information.

Sample number	Diagnosis	Age	Gleason score	Initial PSA
PCa1	PCa	70	7	63.54
PCa2	PCa	73	6	14.3
PCa3	PCa	74	8	65
PCa4	PCa	59	7	26.45
PCa5	PCa	71	9	4.5
PCa6	PCa	62	8	349.6
PCa7	PCa	69	9	21.28
PCa8	PCa	81	7	8.4
PCa9	PCa	67	9	37.21
PCa10	PCa	58	7	12.34
PCa21	PCa	73	7	26.23
BPH1	BPH	51	-	5.06
BPH2	BPH	58	-	1.24
BPH3	BPH	68	-	8.01
BPH4	BPH	63	-	2.75
BPH5	BPH	75	-	3.41
BPH6	BPH	80	-	2.1
BPH7	BPH	73	-	12.35
BPH8	BPH	77	-	0.92
BPH9	BPH	65	-	4.39
BPH10	BPH	68	-	2.53
